# QuEChERS-气相色谱-质谱法检测鱼肉中19种氯酚类化合物

**DOI:** 10.3724/SP.J.1123.2021.12002

**Published:** 2022-05-08

**Authors:** Yinghua MU, Jiali XING, Jian SHEN, Lu YING, Lingyan MAO, Xiaorong XU, Yongjiang LOU, Xi WU

**Affiliations:** 1.宁波大学食品与药品学院, 浙江 宁波 315211; 1. College of Food and Pharmaceutical Sciences, Ningbo University, Ningbo 315211, China; 2.宁波市产品食品质量检验研究院(宁波市纤维所), 浙江 宁波 315048; 2. Ningbo Academy of Product and Food Quality Inspection (Ningbo Fibre Inspection Institute), Ningbo 315048, China

**Keywords:** 气相色谱-质谱, QuEChERS, 氯酚类化合物, 鱼肉, gas chromatography-mass spectrometry (GC-MS), QuEChERS, chlorophenols, fish

## Abstract

大量含氯农药、次氯酸消毒水以及水产品杀虫剂和杀菌剂的广泛使用,使鱼类容易受到氯酚类化合物的污染,因而建立鱼肉中氯酚类化合物的检测方法十分重要。建立了QuEChERS结合气相色谱-质谱法同时检测鱼肉中19种氯酚类化合物的分析方法。19种氯酚类化合物选用DB-5MS毛细管色谱柱(30 m×0.25 mm×0.25 μm),载气流速1 mL/min进行分离,可以得到很好的峰形。前处理采用改良的QuEChERS方法,通过对提取剂的种类和剂量、净化剂的种类和剂量,以及衍生条件中的衍生温度、衍生时间和衍生剂用量等进行优化,确定最优的前处理方法。选择10 mL乙酸乙酯作为提取剂,500 mg的C_18_作为净化剂,加入3 g氯化钠和5 g无水硫酸镁,过0.22 μm的有机滤膜,加入50 μL的硅烷化衍生剂在45 ℃条件下衍生30 min,用EI源测定,选择离子监测模式,以外标法定量。19种氯酚类化合物在0.4~10 μg/L范围内具有良好的线性关系,相关系数*R*^2^大于0.998,方法定量限为0.04~0.16 μg/kg。空白基质不同加标水平的回收率为70.6%~115.0%,相对标准偏差为2.6%~10.5%。将建立的方法应用于实际样品的检测分析,结果显示,各种鱼肉中均有不同程度的氯酚类化合物检出,其中,黄花鱼检出的氯酚类化合物总量最大,为8.74 μg/kg;其次为鲫鱼7.59 μg/kg;米鱼的检出量最少,为1.59 μg/kg。所建立的方法简化了样品的前处理步骤,操作简单,方法灵敏度高、重复性好,可满足鱼肉中19种氯酚类化合物的高通量检测要求,能显著提高氯酚类化合物的检测效率。

## Abstract (en)

With the increasing use of chlorine-containing pesticides, hypochlorous acid disinfection water as well as aquatic product insecticides and fungicides are widely used in the cultivation of fish. This has led to the contamination of fish by chlorophenol compounds. However, currently, there is no standard method for the simultaneous determination of 19 chlorophenol compounds in fish.

In this study, the optimum chromatography and mass spectrometry conditions were determined by investigating the instrument parameters. The 19 chlorophenol compounds were well separated using the DB-5MS capillary chromatographic column (30 m×0.25 mm×0.25 μm) with a carrier gas flow rate of 1 mL/min. Under this condition, the chromatographic peak was sharp and symmetric. An analytical method was developed for the simultaneous determination of the 19 chlorophenol compounds in fish using gas chromatography-mass spectrometry coupled with QuEChERS pretreatment. The improved QuEChERS method was used in sample preparation. The 19 chlorophenol compounds were extracted with organic solvents and purified with purifying agents. During the experiment, the effect of the kinds and volumes of the extraction solvent, as well as the types and dosages of the purifying agent, on the recoveries of the 19 chlorophenol compounds were investigated. Moreover, the temperature and time of derivatization, as well as the dosage of the derivatization agent, were optimized. All aforementioned analyses were conducted with the aim of determining the optimal pretreatment method. Finally, the optimized gas chromatography-mass spectrometry conditions were employed for the quantitative determination of 19 chlorophenol compounds in fish samples. Based on the experimental results, the best extraction method was determined to be the one where the extraction agent (10 mL ethyl acetate) was added to 3 g sodium chloride and 5 g anhydrous magnesium sulfate in the test tube, followed by ultrasonication for 15 min. The sample was centrifuged at 4500 r/min for 5 min, and 500 mg C_18_ was selected as the purifying agent to purify the supernatant. The purified supernatant was blown with nitrogen to less than 1 mL at 45 ℃, and then redissolved with ethyl acetate to 1 mL. Subsequently, the sample solution was passed through a 0.22 μm organic filter membrane, following which 50 μL bis(trimethylsilyl)trifluoroacetamide was added for derivatization at 45 ℃ for 30 min. Lastly, the 19 chlorophenol compounds were determined by gas chromatography-mass spectrometry with an electrospray ionization source and selecting ion monitoring mode. The 19 chlorophenol compounds were then quantitatively analyzed by the external standard method. The compounds showed good linearity in the concentration range of 0.4-10 μg/L, with correlation coefficients (*R*^2^) greater than 0.998. The limits of detection and limits of quantification were 0.01-0.05 μg/kg and 0.04-0.16 μg/kg, respectively. Moreover, the average recoveries of the 19 chlorophenol compounds were in the range of 70.6%-115.0% at three spiked levels, and the relative standard deviations were in the range of 2.6%-10.5%. The established method in this study was applied to detect and analyze chlorophenol compounds in actual samples. The experimental results showed that various levels of chlorophenol compounds could be detected in different fishes. Among them, the total amount of chlorophenol compounds detected in the Corvina was 8.74 μg/kg, followed by the Crucian carp at 7.59 μg/kg, and the minimum detected amount in rice fish (1.59 μg/kg). With its simple operation, high sensitivity, and good repeatability, the established method simplifies the pre-treatment of fish samples. It can also meet the requirements for the high-throughput detection of 19 chlorophenol compounds in fish, thereby significantly improving the detection efficiency of chlorophenols. Moreover, the method provides crucial technical support and a theoretical basis for the establishment of feasible detection standards for chlorophenols in China, as well as for the control of residue levels of chlorophenol compounds in fish. The findings have important practical significance to implement management measures during fish breeding and transportation.

氯酚类化合物(chlorophenols, CPs)是苯环上活性位点的氢原子被氯原子取代后的产物,根据所取代的氯原子数不同可分为单氯酚(chlorophenol, CP)、二氯酚(dichlorophenol, DCP)、三氯酚(trichlorophenol, TrCP)、四氯酚(tetrachlorophenol, TeCP)和五氯酚(pentachlorophenol, PCP)共19种同分异构体^[1]^,其中单氯酚和四氯酚共有3种同分异构体,二氯酚和三氯酚共有6种同分异构体。CPs是木材、纺织品、皮革制品及纸制品的防霉防腐剂,是果树、蔬菜及其他农作物和水产品的杀虫剂和杀菌剂,以及农作物的除草剂^[2]^。大量研究表明,CPs具有致畸、致癌和致突变作用,且其毒性随着氯原子数目的增多而增强,间位氯取代基的毒性明显高于邻位氯取代基^[3⇓-5]^。CPs种类多、危害大、不易降解,低浓度的CPs便可带来严重的水质污染,并可通过生物富集在鱼体内达到很高的浓度,进而给人类带来安全隐患,已成为重点监控的环境污染物之一,现已被许多国家禁止使用。如美国环境保护署(environmental protection agency, EPA)与人类环境健康评估组根据对2,4,5-三氯酚可能的致肿瘤效应的研究将其归为D等级致癌物^[6]^。此外,2-氯酚、2,4-二氯酚、2,4,6-三氯酚和五氯酚已经被EPA列为优先控制污染物^[7]^。因而有必要对水环境和其中的生物体进行监测。但目前对生物体中氯酚的检测标准仅见于五氯酚或五氯酚钠盐的测定^[8⇓-10]^,国内外尚无水生生物体中涵盖19种CPs同时检测的方法标准和限量标准。

现有文献报道的CPs的测定方法多为色谱法,主要有气相色谱法(GC)^[11,12]^、气相色谱-质谱法(GC-MS)^[13⇓-15]^、液相色谱法(LC)^[16]^、液相色谱-质谱法(LC-MS)等^[17⇓-19]^。LC和LC-MS不需要衍生化处理且检测较快,但其定性能力和灵敏度较低,对溶剂要求较高,分析成本和日常维护费用较高。GC和GC-MS都需将CPs进行衍生化处理再进行分析,但GC-MS灵敏度更高,定性更可靠,相比于LC-MS检测成本更低,被广泛应用于CPs的测定。目前对于环境样品、纺织品以及皮革制品和纸制品中多种CPs同时测定的方法已有文献报道,如王成云等^[20]^建立了GC-MS测定纸和纸制品中19种CPs的方法,各组分的定量限均为2 μg/kg。莫贤科等^[21]^建立了GC-MS测定皮革中19种CPs的方法,前处理方法较为复杂,但也能满足皮革中CPs的日常检测。

目前对鱼肉中氯酚的检测多见于三氯酚、四氯酚、五氯酚或五氯酚钠盐的测定^[22]^,而19种CPs同时检测的方法报道较少。钟惠英等^[23]^建立了GC-MS法测定大黄鱼和草鱼中的19种CPs,前处理采用硫酸消解后的液液萃取,较为复杂,且消耗大量有机溶剂。近年来发展较快的QuEChERS简化了样品处理过程,且对环境污染较小,具有高效、经济、快速、简便等优点,被广泛应用于有机污染物残留检测的前处理中^[24,25]^。如Padilla-Sanchez等^[26]^采用QuEChERS前处理结合气相色谱-三重四极杆质谱法对农用土壤中的2-氯酚、4-氯酚、2,4-二氯酚、2,4,6-三氯酚、2,4,5-三氯酚和五氯酚进行了检测,实现了样品的快速前处理,节省了检测时间。王连珠等^[27]^建立了QuEChERS结合UPLC-MS/MS测定动物源食品(猪肉、猪肝、鸡肉、鱼肉、牛奶、鸡蛋)中痕量五氯酚。黄建芳^[28]^采用QuEChERS结合HPLC-MS/MS测定大黄鱼中五氯酚的含量,检出限为0.1 μg/kg,但该方法只对五氯酚进行检测,未对其他氯酚类化合物的同分异构体进行分析。

鉴于当前鱼中19种CPs同时检测的方法较少,现有的研究存在前处理复杂或仅能检测少数几种CPs的现状,因此本研究开发了基于改良的QuEChERS前处理提取净化方法,结合GC-MS技术对鱼中19种CPs同时测定,通过化合物的色谱保留时间和峰面积进行定性和定量分析,并通过实际样品进行了方法的验证。建立的方法能够减少分析时间和溶剂消耗、降低环境污染、提高检测效率,可适用于鱼肉中19种CPs的快速检测。

## 1 实验部分

### 1.1 仪器、试剂与材料

TSQ 8000 Evo气相色谱-三重四极杆质谱联用仪(美国安捷伦科技有限公司); DB-5MS毛细管色谱柱(30 m×0.25 mm×0.25 μm);分析天平(中国北京赛多利斯科学仪器有限公司);高速离心机(美国Thermo Fisher Scientific公司); Vortex 3自动漩涡混合器(德国IKA公司);一次性无菌注射器(中国上海金塔医用器材有限公司); Milli-Q Academic超纯水仪(美国Millipore公司); KQ-500E超声波清洗仪(中国宁波海曙科盛超声设备有限公司);多功能涡旋混合器(德国IKA公司);多功能全自动氮吹浓缩仪(瑞典Biotage公司);移液枪(法国GILSON公司); 0.22 μm滤膜(美国Waters公司)。

实验用的鱼类(带鱼、鲫鱼、鲈鱼、鲢鱼、鲤鱼、草鱼等)购于宁波路林市场。乙腈、丙酮(色谱纯),购自德国Merck公司;正己烷、乙酸乙酯(色谱纯),购自中国天津致远化学试剂有限公司;二氯甲烷、环己烷(色谱纯),购自中国江苏永华化学科技有限公司;无水硫酸镁、氯化钠(分析纯),购自中国国药化学试剂有限公司;十八烷基键合硅胶(octadecylsilane chemically bonded silica, C_18_)、中性氧化铝(alumina-N, AL-N)、石墨化炭黑(graphitized carbon black, GCB)、乙二胺-*N*-丙基硅烷(primary secondary amine, PSA),硅烷化衍生试剂:*N*,*O*-双(三甲基硅烷)三氟乙酰胺(bis(trimethylsilyl)trifluoroacetamide, BSTFA),购自中国上海安谱实验科技股份有限公司。

氯酚混合标准溶液:一氯酚(2-CP、3-CP、4-CP),二氯酚(2,3-DCP、2,4-DCP、2,5-DCP、2,6-DCP、3,4-DCP、3,5-DCP),三氯酚(2,3,4-TrCP、2,3,5-TrCP、2,3,6-TrCP、2,4,5-TrCP、2,4,6-TrCP、3,4,5-TrCP),四氯酚(2,3,4,5-TeCP、2,3,4,6-TeCP、2,3,5,6-TeCP),五氯酚(PCP)(上海安谱实验科技股份有限公司),质量浓度均为1000 μg/mL,使用时用正己烷稀释至所需浓度。

### 1.2 标准溶液的配制

氯酚标准储备液的配制:原混合标准溶液质量浓度为1000 μg/mL,溶于二氯甲烷,使用时用正己烷配制。准确量取100 μL原混合标准溶液液于10 mL的容量瓶中,用正己烷定容至刻度线,摇匀,配制成10 μg/mL的储备液,置于4 ℃的冰箱中保存备用。

混合标准溶液系列梯度的配制:吸取10 μg/mL的混合标准储备液10 μL于1 mL的进样瓶中,用正己烷定容至刻度线,摇匀,配制成100 μg/L的中间液,依次取100 μg/L的中间液10、20、40、60、80、100 μL于6个2 mL的进样瓶中,分别用正己烷定容至1 mL,密封、摇匀。再取10 μg/L的溶液40、80 μL用正己烷定容至1 mL,密封、摇匀。得到标准系列溶液,质量浓度依次为0.4、0.8、1、2、4、6、8、10 μg/L。

### 1.3 样品的制备及其前处理

提取:选取鱼样的肌肉组织绞碎均质,置于-18 ℃的冰箱中保存,准确称取5.00 g鱼肉样品于50 mL的聚乙烯塑料离心管中,加入5 mL超纯水和10 mL乙酸乙酯,涡旋分散30 s,然后向管中加入3 g氯化钠和5 g无水硫酸镁,涡旋振荡1 min,随后40 ℃超声15 min,再以4500 r/min离心5 min。

净化:收集离心好的上清液至装有500 mg的C_18_和100 mg无水硫酸镁的15 mL聚乙烯塑料离心管中,涡旋振荡30 min,超声15 min,高速离心后取5 mL上清液于另一15 mL试管中,在45 ℃水浴中氮吹浓缩至小于1 mL,再用乙酸乙酯定容至1 mL,然后过0.22 μm的有机滤膜后再加入50 μL衍生试剂,在45 ℃衍生30 min,最后摇匀、冷却至室温,用GC-MS进行上机检测。

### 1.4 仪器分析条件

GC条件:DB-5MS毛细管色谱柱(30 m×0.25 mm×0.25 μm);进样口温度为:260 ℃;载气为高纯He(纯度≥99%);流速为1.0 mL/min。程序升温条件:初始温度70 ℃,维持1 min;然后以8 ℃/min升至160 ℃;再以3 ℃/min升至200 ℃;最后以40 ℃/min升至300 ℃,保持3 min。不分流进样;进样量为1 μL。

MS条件:离子源温度260 ℃,传输线温度280 ℃, EI离子源,电子能量:70 eV,溶剂延迟10 min,数据采集采用全扫模式定性,选择离子监测(SIM)模式定量,19种CPs的监测离子详见表1。

**表1:** 19种CPs的保留时间、定量、定性离子

Chlorophenol	Retention time/min	Quantitation ion (m/z)	Identification ions (m/z)
2-Chlorophenol (2-CP)	8.04	185	185, 187, 200
3-Chlorophenol (3-CP)	8.29	185	185, 187, 200
4-Chlorophenol (4-CP)	8.16	185	185, 187, 200
2,5-Dichlorophenol (2,5-DCP)	10.50	219	93, 219, 234
2,6-Dichlorophenol (2,6-DCP)	10.54	219	93, 219, 234
3,5-Dichlorophenol (3,5-DCP)	10.55	219	93, 219, 234
2,4-Dichlorophenol (2,4-DCP)	10.80	219	93, 219, 234
2,3-Dichlorophenol (2,3-DCP)	11.21	219	93, 219, 234
3,4-Dichlorophenol (3,4-DCP)	11.39	219	93, 219, 234
2,4,6-Trichlorophenol (2,4,6-TrCP)	12.75	253	253, 255, 268
2,3,5-Trichlorophenol (2,3,5-TrCP)	13.05	253	253, 255, 268
2,4,5-Trichlorophenol (2,4,5-TrCP)	13.16	253	253, 255, 268
2,3,6-Trichlorophenol (2,3,6-TrCP)	13.34	253	253, 255, 268
3,4,5-Trichlorophenol (3,4,5-TrCP)	13.80	253	253, 255, 268
2,3,4-Trichlorophenol (2,3,4-TrCP)	14.18	253	253, 255, 268
2,3,5,6-Tetrachlorophenol (2,3,5,6-TeCP)	16.10	289	287, 289, 304
2,3,4,6-Tetrachlorophenol (2,3,4,6-TeCP)	16.32	289	287, 289, 304
2,3,4,5-Tetrachlorophenol (2,3,4,5-TeCP)	16.83	289	287, 289, 304
Pentachlorophenol (PCP)	20.62	323	321, 323, 325, 338

## 2 结果与讨论

### 2.1 仪器条件的优化

#### 2.1.1 色谱条件的优化

CPs含羟基,使分子的极性增强,不利于直接色谱分离^[29]^。因此,需要衍生化,变成极性弱的物质,提高其挥发性,减少色谱分离过程中的峰拖尾,便于在色谱柱上分离。国家标准GB/T 18414.2-2006《纺织品含氯苯酚的测定》中采用了DB-17MS具有中等极性的色谱柱^[30]^,但该色谱柱只对TeCP和PCP这两类酚有很好的分离效果,对TrCP分离效果不好,因此本实验参照ISO 17070∶2015(E)《皮革化学测试测定含氯苯酚的含量》^[31]^,采用DB-5MS毛细管色谱柱对19种CPs的衍生化产物进行检测,各同分异构体的出峰顺序及保留时间分别见图1和表1。

**图1:**
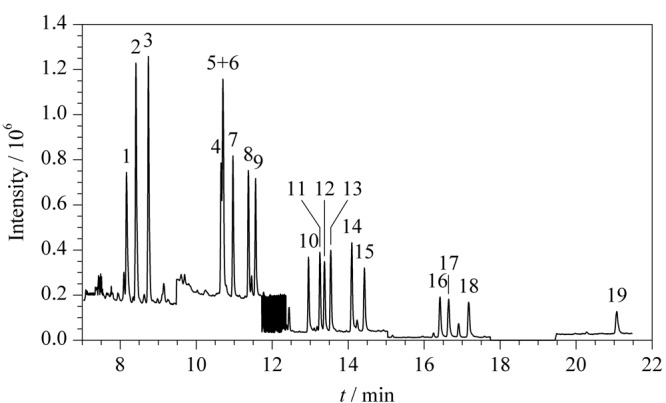
19种CPs的总离子流色谱图

如图1所示,DB-5MS色谱柱除了无法分离2,6-DCP和3,5-DCP外,对其他17种氯酚均能完全分开且峰形较好。这进一步说明了2,6-DCP和3,5-DCP性质相似,因而难以将两者分离。所以应用本方法进行测定时2,6-DCP和3,5-DCP只能测定其二者的总量。

为进一步优化方法,获得更好的检测效率,在上述色谱柱的条件下进行载气流速的优化,选择不同流速0.8、1.0、1.2 mL/min,实验结果发现随着载气流速的降低,2,6/3,5-DCP峰未见很好的分离,但载气流速过慢导致拖尾峰以及分析时间大幅增加。因此,在保证分离度的基础上,综合评估,选取1.0 mL/min的载气流速。

#### 2.1.2 质谱条件的优化

先对19种CPs单标准溶液采用全扫描模式进样,初步确定其对应的特征定量离子及其保留时间,分别找出丰度最高的离子碎片,如表1所示。进一步采用SIM模式,只对待测组分物质离子信号进行定性定量采集,很大程度上提高了对目标化合物的检测灵敏度,减少了杂质的干扰,从而使试验结果更加准确可靠。

### 2.2 前处理方法的优化

#### 2.2.1 提取溶剂种类及用量的优化

不同的提取剂对CPs的提取效果不同,且产生的杂质干扰也不同。为了确定合适的提取溶剂,本实验选择正己烷、环己烷、二氯甲烷、乙酸乙酯进行考察,结果如图2所示。二氯甲烷和乙酸乙酯的回收率相当(64.0%~113.0%和67.0%~116.0%, *p*>0.05),高于弱极性的环己烷和正己烷的回收率(43.8%~85.0%和51.0%~92.0%)。考虑到二氯甲烷沸点较低,在实验操作过程中易挥发,且二氯甲烷易带来杂质峰,不利于大批量样本的测定,因此选取强极性的乙酸乙酯作为提取剂。

**图2:**
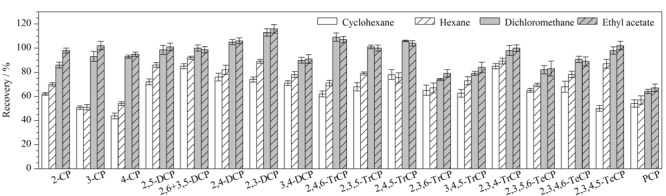
不同提取剂对19种CPs回收率的影响(*n*=6)

同时,本实验还考察了提取剂用量(6、8、10、12 mL)的影响。结果显示,10 mL乙酸乙酯足以完全提取基质中的目标化合物,19种CPs的平均加标回收率达到最佳,在78.0%~112.0%之间;提取溶剂过多不仅会造成试剂浪费,还会导致基质中共萃取干扰物不断增多,从而导致基质效应不断加强;与黄武等^[32]^的实验结果一致(5 g样品选择10 mL提取剂时提取酚类化合物的回收率最高)。因此,本实验最终选择提取剂用量为10 mL。

#### 2.2.2 净化剂种类及用量的优化

鱼肉中含有脂肪、蛋白质、色素等物质,这些物质会干扰19种CPs的离子化,影响其检测分析^[33]^。所以本实验选择C_18_、AL-N、PSA和GCB这4种净化剂,考察净化效果。

由表2可以看出,使用AL-N和GCB时都有3种氯酚的平均回收率低于60.0%,分别是2,4,6-TrCP、2,3,5-TrCP、PCP和2,4,6-TrCP、2,4,5-TrCP、PCP。PSA和C_18_的回收率范围分别是60.5%~103.0%和69.8%~108.0%,但使用PSA时2,4,6-TrCP、2,3,4-TrCP和PCP的回收率较低(64.3%、68.9%和60.5%),使用C_18_时只有PCP的回收率较低(69.8%),其余18种CPs大多集中在82.0%~108.0%之间。可能的原因是,C_18_可以很好地除去疏水性共提物,如脂肪、有机酸等,还能有效除去非极性杂质,而CPs是极性的,所以除杂效果更好,与黄建芳^[28]^的研究结果一致。因此,选择C_18_作为19种CPs的净化剂。

**表2:** 不同净化剂对19种CPs回收率的影响(*n*=6)

Chlorophenol	Average recoveries/%			
	AL-N	PSA	C_18_	GCB
2-CP	98.7	81.6	92.5	85.7
3-CP	106.0	101.0	102.0	102.0
4-CP	87.2	79.8	82.8	77.3
2,5-DCP	76.4	82.5	89.4	68.2
2,6-DCP+3,5-DCP	76.4	76.9	99.8	68.2
2,4-DCP	98.7	101.0	108.0	109.7
2,3-DCP	66.2	93.4	86.7	69.3
3,4-DCP	101.3	83.4	89.0	92.4
2,4,6-TrCP	56.8	64.3	83.5	58.7
2,3,5-TrCP	53.7	81.4	89.4	88.3
2,4,5-TrCP	64.3	82.7	82.0	51.3
2,3,6-TrCP	96.3	72.1	90.2	101.0
3,4,5-TrCP	95.3	101.0	103.5	89.2
2,3,4-TrCP	94.2	68.9	99.3	90.2
2,3,5,6-TeCP	101.9	103.0	106.7	94.4
2,3,4,6-TeCP	91.4	71.1	86.0	88.4
2,3,4,5-TeCP	112.0	87.4	107.9	110.0
PCP	58.0	60.5	69.8	57.8

recoveries of the 19 CPs (*n*=6)

AL-N: alumina-N; PSA: primary secondary amine; C_18_: octadecylsilane chemically bonded silica; GCB: graphitized carbon black.

本实验进一步考察了不同C_18_用量(100、300、500、700 mg)对19种CPs回收率的影响。由图3可以看出,C_18_为500 mg时,目标化合物的回收率相对较高,为76.0%~114.0%,且绝大多数目标物的回收率都集中在89.2%~114.0%之间,因此选为所用。

**图3:**
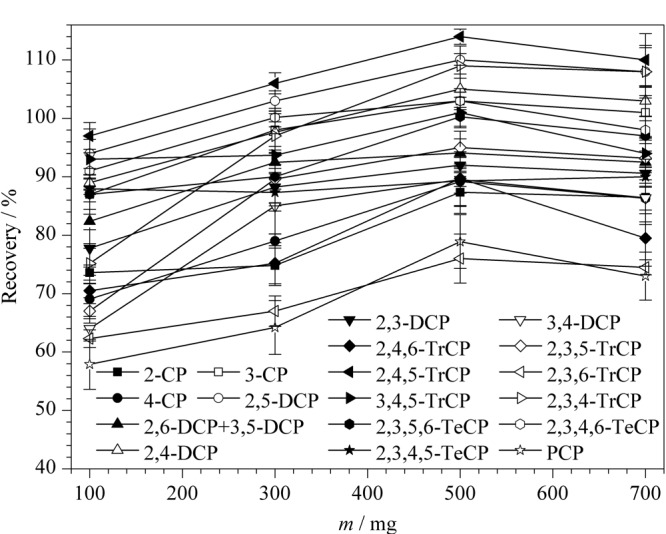
不同C_18_用量对19种CPs回收率的影响(*n*=6)

#### 2.2.3 衍生条件的优化

硅烷化衍生具有热稳定性好、挥发性强、易于制备以及色谱性能好等优点^[34]^。CPs极性强,挥发性和稳定性都较低,不适于直接进样。为获得良好的峰形,需对提取物进行衍生化处理,因此本实验选用BSTFA进行硅烷化衍生。考察了不同衍生剂体积对19种CPs回收率的影响(见表3)。CPs的回收率随着衍生剂添加量的增加而增加,当达到50 μL时,衍生剂与目标物的反应达到饱和,回收率达到最佳,其回收率范围为68.9%~115.0%;当衍生剂添加量为60 μL时,回收率基本保持不变;到70 μL时略有下降,回收率范围为67.6%~109.0%。因此本实验选择衍生剂的量为50 μL。

**表3:** 不同衍生剂体积对19种CPs回收率的影响(*n*=6)

Chlorophenol	Average recoveries/%				
	30 μL	40 μL	50 μL	60 μL	70 μL
2-CP	80.7	87.1	86.5	87.0	85.0
3-CP	92.2	100.7	101.0	99.5	97.8
4-CP	72.4	82.6	81.5	82.0	80.0
2,6-DCP+3,5-DCP	84.5	91.4	90.3	89.0	86.5
2,5-DCP	89.7	101.0	102.0	101.0	98.6
2,4-DCP	99.1	102.0	103.0	101.0	98.4
2,3-DCP	89.3	98.6	115.0	112.0	109.0
3,4-DCP	83.7	96.4	112.0	109.0	105.0
2,6-DCP	76.0	93.2	98.4	98.5	97.2
2,4,6-TrCP	79.0	87.1	95.4	96.0	93.0
2,3,5-TrCP	86.5	98.4	101.0	99.6	98.7
2,4,5-TrCP	76.0	88.4	97.8	98.0	87.6
2,3,6-TrCP	85.2	98.6	106.0	104.0	101.0
3,4,5-TrCP	83.7	89.8	96.7	97.0	92.4
2,3,4-TrCP	89.5	96.8	108.0	107.0	105.0
2,3,5,6-TeCP	84.3	96.8	99.6	97.7	96.2
2,3,4,6-TeCP	99.8	100.4	106.0	104.0	101.0
2,3,4,5-TeCP	78.8	87.4	93.6	94.0	92.1
PCP	62.3	65.4	68.9	68.7	67.6

the recoveries of the 19 CPs (*n*=6)

随后对衍生温度(35、40、45、50 ℃)进行了优化,实验结果发现,随着衍生温度的上升,回收率逐渐呈上升趋势,达到45 ℃时趋于平缓。在45 ℃时平均回收率达到最佳,为94.6%,所以本实验选用衍生温度为45 ℃。

在45 ℃下,加入50 μL衍生剂,分别衍生20、30、40、50 min。结果发现,在20 min时衍生化反应不完全,目标物的回收率在49.0%~110.0%之间;在30 min时衍生化反应完全,19种CPs的回收率在74.0%~112.0%之间;在40 min和50 min时,其回收率效果差异不大。所以本实验选择衍生30 min以提高衍生效率。

### 2.3 方法学评价

#### 2.3.1 线性范围与方法的检出限

以空白基质提取液为溶剂,配制19种CPs混合标准溶液,在优化的色谱条件下进样检测。以氯酚化合物的质量浓度(*x*, μg/L)为横坐标、峰面积(*y*)为纵坐标,绘制校准曲线,计算回归方程。结果如表4所示,19种CPs的相关系数*R*^2^均大于0.998,在0.4~10 μg/L范围内线性关系良好。同时,在最低添加水平下,以3倍信噪比结合浓度外推法确定方法的检出限(limit of detection, LOD),以10倍信噪比确定方法的定量限(limit of quantification, LOQ)。

**表4:** 19种CPs的线性范围、线性方程、相关系数、检出限和定量限

Chlorophenol	Linear range/(μg/L)	Linear equation	R^2^	LOD/(μg/kg)	LOQ/(μg/kg)
2-CP	0.4-10	y=7.758×10^4^x+1.023×10^4^	0.9990	0.02	0.07
3-CP	0.4-10	y=1.549×10^5^x+2.241×10^4^	0.9990	0.05	0.15
4-CP	0.4-10	y=1.427×10^5^x-9.968×10^3^	0.9995	0.03	0.10
2,5-DCP	0.4-10	y=5.411×10^4^x-6.486×10^2^	0.9997	0.01	0.04
2,6-DCP+3,5-DCP	0.4-10	y=1.719×10^5^x+3.156×10^4^	0.9983	0.01	0.04
2,4-DCP	0.4-10	y=5.162×10^4^x-2.254×10^3^	0.9997	0.02	0.06
2,3-DCP	0.4-10	y=5.653×10^4^x-4.791×10^3^	0.9993	0.02	0.08
3,4-DCP	0.4-10	y=7.47×10^4^x-3.027×10^2^	0.9997	0.01	0.04
2,4,6-TrCP	0.4-10	y=3.203×10^4^x-2.479×10^3^	0.9996	0.04	0.14
2,3,5-TrCP	0.4-10	y=4.044×10^4^x-4.319×10^3^	0.9990	0.03	0.12
2,4,5-TrCP	0.4-10	y=3.232×10^4^x+1.416×10^3^	0.9992	0.04	0.14
2,3,6-TrCP	0.4-10	y=3.974×10^4^x-3.531×10^3^	0.9993	0.03	0.10
3,4,5-TrCP	0.4-10	y=4.474×10^4^x-1.513×10^3^	0.9998	0.01	0.04
2,3,4-TrCP	0.4-10	y=3.388×10^4^x-4.986×10^2^	0.9996	0.05	0.16
2,3,5,6-TeCP	0.4-10	y=2.850×10^4^x-1.921×10^3^	0.9993	0.04	0.12
2,3,4,6-TeCP	0.4-10	y=2.732×10^4^x-2.681×10^3^	0.9991	0.02	0.07
2,3,4,5-TeCP	0.4-10	y=2.845×10^4^x-2.113×10^3^	0.9992	0.04	0.15
PCP	0.4-10	y=1.361×10^4^x-7.501×10^2^	0.9991	0.05	0.16

y: peak area; x: mass concentration, μg/L.

#### 2.3.2 回收率与精密度

以空白鱼样为基质,分别添加低、中、高3个不同加标水平(0.16、0.32、1.6 μg/kg)的混合标准溶液,然后按优化的样品前处理方法进行测定,计算回收率和测定值的相对标准偏差,每个加标水平平行测定6次,评估该方法的准确性和精密度,结果详见表5。各组分的平均加标回收率为70.6%~115.0%,精密度(RSD)为2.6%~10.5%(*n*=6)。

**表5:** 19种CPs的加标回收率和相对标准偏差(n=6)

Chlorophenol	Low level (0.16 μg/kg)			Medium level (0.32 μg/kg)			High level (1.6 μg/kg)	
	Recovery/%	RSD/%		Recovery/%	RSD/%		Recovery/%	RSD/%
2-CP	76.5	8.7		78.7	6.8		75.0	7.5
3-CP	94.0	8.9		96.5	7.6		86.4	6.8
4-CP	82.0	7.6		86.4	6.2		82.7	4.3
2,5-DCP	93.0	6.8		97.0	5.7		107.0	7.6
2,6-DCP+3,5-DCP	98.5	6.5		102.0	7.4		114.0	8.2
2,4-DCP	109.0	9.4		103.0	10.5		97.0	7.3
2,3-DCP	76.0	10.5		79.4	7.1		82.0	6.2
3,4-DCP	102.0	5.5		96.0	4.2		98.5	4.7
2,4,6-TrCP	79.0	7.5		82.0	3.5		83.0	5.6
2,3,5-TrCP	96.5	3.5		98.0	6.2		95.0	5.2
2,4,5-TrCP	97.2	3.6		94.6	4.5		103.8	4.3
2,3,6-TrCP	80.4	2.6		88.7	2.8		94.2	7.4
3,4,5-TrCP	93.6	6.2		95.7	3.6		102.0	3.4
2,3,4-TrCP	96.5	8.3		84.2	3.2		88.7	4.6
2,3,5,6-TeCP	79.6	5.4		76.5	4.3		86.0	6.6
2,3,4,6-TeCP	89.6	4.2		87.5	5.4		95.1	6.1
2,3,4,5-TeCP	102.0	7.1		103.0	7.3		115.0	2.9
PCP	74.9	9.5		70.6	3.7		73.5	5.7

#### 2.3.3 基质效应

本实验通过比较带鱼、鲤鱼、黄花鱼空白基质溶液配制的混合标准溶液与正己烷配制的混合标准溶液的斜率来评价基质效应(matrix effect, ME)。

计算公式为:ME=[(基质匹配标准曲线斜率/溶剂标准曲线斜率)-1]×100%。ME为负值表示存在基质抑制效应,正值表示存在基质增强效应。以其绝对值为判断依据,绝对值越大则基质效应越强^[35⇓-37]^。当|ME|<20%,为弱基质效应,可忽略;当20%≤|ME|≤50%,为中等强度基质效应;当|ME|>50%,为强基质效应^[38,39]^。结果如表6所示,带鱼中除2,5-DCP表现为强基质增强效应,其他化合物均为弱或中等强度基质效应;黄花鱼中除2,3-DCP、2,3,5,6-TeCP和2,3,4,6-TeCP表现为强基质抑制效应,其他均为弱或中等强度基质效应;鲤鱼均表现为弱基质效应或中等基质效应。因此,本方法采用基质匹配标准溶液进行校正,以消除基质带来的影响,改善回收率。

**表6:** 不同鱼类样品中19种CPs的基质效应

Chlorophenol	Matrix effects/%		
	Hairtail	Corvina	Cyprinoid
2-CP	16.8	-5.7	7.4
3-CP	-7.5	-6.3	11.7
4-CP	-11.9	-13.2	-7.5
2,5-DCP	73.0	17.6	16.9
2,6-DCP+3,5-DCP	34.8	-21.2	1.8
2,4-DCP	13.8	-16.4	19.8
2,3-DCP	26.4	-70.0	34.5
3,4-DCP	21.2	17.3	33.2
2,4,6-TrCP	9.2	-21.7	17.4
2,3,5-TrCP	-1.0	-24.1	1.1
2,4,5-TrCP	9.7	-12.5	19.3
2,3,6-TrCP	14.0	-28.0	38.0
3,4,5-TrCP	-5.2	-13.0	5.5
2,3,4-TrCP	14.3	-20.0	26.4
2,3,5,6-TeCP	15.7	-80.0	-3.4
2,3,4,6-TeCP	-20.9	-73.0	-13.8
2,3,4,5-TeCP	11.0	-40.7	20.0
PCP	-27.3	-37.6	-28.4

#### 2.3.4 与其他方法的比较

将本研究与近几年文献报道的检测CPs的前处理方法进行了简单对比(见表7)。本方法与符昌雨等^[22]^的超声萃取(ultrasonic extraction, UE)结合GC-MS检测水产品中10种CPs的方法相比,可以实现19种CPs的同时检测。UE虽操作简单,但净化不完全,导致检测灵敏度降低,如黄姣等^[40]^和陈秋凯等^[41]^的方法检出限为20 μg/kg。与钟惠英等^[23]^、莫贤科等^[21]^的液液萃取(liquid-liquid extraction, LLE)结合GC-MS方法相比,具有前处理简便、消耗溶剂少、提取效率高等优点。固相萃取(solid phase extraction, SPE)具有净化效果好、灵敏度高、重现性好等优点,但处理步骤较复杂,本文的QuEChERS方法较为简单,避免了SPE方法繁琐的活化、上样、淋洗和洗脱过程,样品提取净化成本低。结合GC-MS进行检测,进一步提高了检测结果的准确度和灵敏度。

**表7:** 本文方法与其他文献方法的比较

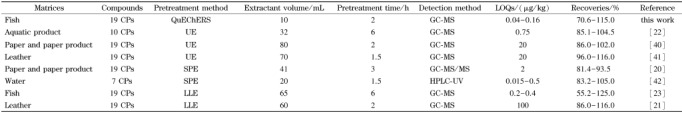

#### 2.3.5 实际样品的检测

应用本方法分别对带鱼、巴沙鱼、鲤鱼和黄花鱼等15个样品进行检测,结果如表8所示,在7个样品中同时检测出2,4-DCP、3,4-DCP、2,3,4-TrCP、2,3,4,5-TeCP,其中最小检出值为0.04 μg/kg,最大检出值为4.8 μg/kg。15个鱼样中黄花鱼检出的CPs总量最大,为8.74 μg/kg,其次为鲫鱼7.59 μg/kg,米鱼的检出量最少,为1.59 μg/kg。

**表8:** 19种CPs的实际样品检测结果

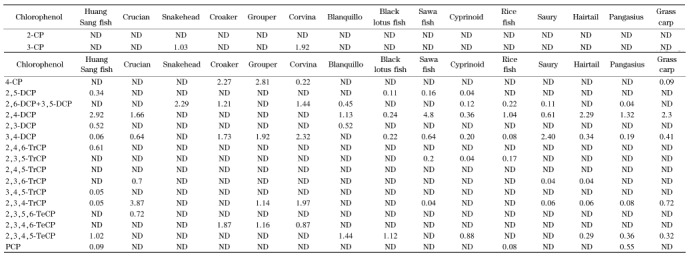

## 3 结论

本研究建立了QuEChERS-气相色谱-质谱测定鱼肉样品中19种CPs含量的方法。通过优化前处理条件以及衍生条件,该方法在0.4~10 μg/L范围内呈良好的线性关系,线性相关系数均大于0.998,方法检出限为0.01~0.05 μg/kg。应用本方法进行实际样品检测,不同鱼肉样品中均有不同程度的氯酚类污染物检出。其中,黄花鱼和鲫鱼污染最严重,石斑鱼和大黄鱼次之,米鱼污染程度最轻。所建立的方法简化了样品的前处理步骤,操作简单,方法灵敏度高、重复性好,可满足鱼肉中19种CPs的高通量检测要求,显著提高CPs的检测效率。
